# Predicting survival in patients admitted to intensive care following out-of-hospital cardiac arrest using the Prognosis After Resuscitation score

**DOI:** 10.1186/cc13681

**Published:** 2014-03-17

**Authors:** H Davies, A Loosely, S Dolling, R Eve

**Affiliations:** 1 University Hospitals Bristol NHS Foundation Trust, Bristol, UK

## Introduction

Out-of-hospital cardiac arrest (OHCA) has a poor prognosis even after successful resuscitation [1]. No scoring system has been fully validated for predicting survival following OHCA. The Prognosis After Resuscitation (PAR) score was developed from metaanalysis in 1992. A score >5 (from seven variables) predicts nonsurvival following in-hospital cardiac arrest (IHCA) [2,3]. Porter and colleagues demonstrated that PAR >5 is a useful predictor of nonsurvival for combined IHCA/OHCA ICU admissions [4]. We aim to evaluate PAR application to adult ICU admissions at University Hospitals Bristol (UHB), UK following OHCA.

## Methods

The Innovian (Dräger) electronic clinical information database was searched retrospectively for all OHCA ICU admissions from 2010 to 2012. Data were extracted using an electronic *pro forma *in Microsoft Excel. Missing/incomplete data were excluded. Survival was defined as survival to discharge from the acute hospital (UHB).

## Results

There were 247 admissions to the ICU following OHCA from 2010 to 2012. Seven patients were excluded for missing/incomplete data. In total, 102 patients (42.5%) survived to discharge. PAR ranged from -2 to 18. Zero was the most frequent score. Only one of 15 admissions with PAR >5 (PAR 13) survived. A total of 101 patients with PAR ≤5 survived (45%). Acute myocardial infarction was identified as the precipitating event in 111 patients (46%).

## Conclusion

Only one of 240 OHCA patients admitted to the ICU over a 3-year period with PAR >5 survived until discharge. PAR scoring has been shown to be useful in helping decide the appropriateness of ICU admission for combined IHCA/OHCA. Our data support its use in isolated OHCA. PAR ≤5 appears to be poorly predictive of survival. However, PAR >5, combined with clinical evaluation, could help identify OHCA nonsurvivors and avoid ICU admissions that do not benefit the patient.
